# Experience of Vulnerable Women Narrated through the Body-Mapping Technique

**DOI:** 10.3390/ijerph182413094

**Published:** 2021-12-11

**Authors:** Jacqueline de Souza, Carla Aparecida Arena Ventura, Jordana Luiza Gouvêa de Oliveira, Loraine Vivian Gaino, Juliana Cristina dos Santos Monteiro, Jaqueline Lemos de Oliveira, Leticia Yamawaka de Almeida, Solina Richter, Denise Saint-Arnault

**Affiliations:** 1 Department of Psychiatric Nursing and Human Sciences, Ribeirão Preto College of Nursing, University of São Paulo, Ribeirão Preto 14040-902, Brazil; jacsouza2003@usp.br (J.d.S.); caaventu@eerp.usp.br (C.A.A.V.); jordanaluiza.oliveira@gmail.com (J.L.G.d.O.); lorainegaino@gmail.com (L.V.G.); jumonte@eerp.usp.br (J.C.d.S.M.); leyamawaka@gmail.com (L.Y.d.A.); 2 College of Nursing, Saskatoon Campus Health Sciences, University of Saskatchewan, Saskatoon, SK S7N 5E5, Canada; mrichter@ualberta.ca; 3 School of Nursing, University of Michigan, Ann Arbor, MI 48109, USA; starnaul@med.umich.edu

**Keywords:** mental health, stress, women

## Abstract

Vulnerable women are considered a priority in public policies and research agendas. It is necessary to understand better the specificities of their daily lives and the meanings they attribute to their experiences, as this undoubtedly contributes to more grounded and culturally appropriate practices. Additionally, innovative techniques in qualitative research are demanded in academia. This narrative research study was carried out with fourteen women from a Brazilian socioeconomically vulnerable neighborhood. We used the body-mapping technique to investigate the experiences of women with mental health disorders or psychosocial distress. The aim was to analyze the self-perception about daily stressors and discuss the feasibility of this technique to facilitate this group’s storytelling. Data collection was performed through focus groups, guided by the body-mapping technique steps, and supplemented with individual interviews. Interpersonal conflicts and violence were the main stressors. These strongly impacted the well-being of these women and their children. Some important personal qualities and resilience were identified. Body-mapping played a fundamental role in facilitating storytelling. It amplified the linguistic possibilities for participants to express their feelings and promoted reflections about the present, past, and glimpses into the future.

## 1. Introduction

Social and health agencies have highlighted the female gender as one of the priority groups in policies, programs, and research [1]. Furthermore, considering the necessity of developing more effective and culturally appropriate interventions, it is strongly recommended to consider the meaning women themselves attribute to their experiences [2].

In this sense, it is necessary to emphasize the specificities of vulnerable women and the importance of studies that broaden the understanding of the stressors, their causes, and their effects on this specific group [3,4].

There are many recent studies developed with vulnerable women in different cultures. In general, researchers have started from a set of stressors previously described in the literature, such as socioeconomic issues [5,6], natural disasters [7], violence [8,9,10], depression [11,12], pregnancy and postpartum [13,14], racism [15], and gender inequality [1]. These studies used different methodological approaches. However, there is a tendency to use more traditional data collection techniques such as surveys and interviews. Therefore, a gap is identified in the direction of the development of studies in specific cultural contexts and whose methodological approaches and techniques explore different forms of language (oral, visual, and artistic) as a means of giving voice to vulnerable women to better report, from their points of view, the elements they perceive as stressors in their daily lives. Thus, one of the innovative aspects of our study was the use of the body-mapping technique [16] to investigate the experiences of women with mental health disorders or psychosocial distress living in a Brazilian socioeconomically vulnerable neighborhood based on the question: “What is the perception of these vulnerable women about their daily life stressors?”. Thus, this study aimed to analyze the perception of these vulnerable women about their daily stressors and discuss the feasibility of the body-mapping technique to facilitate storytelling in this group.

The body-mapping technique is a strategy based on the assumption that one’s lived experiences are strongly marked within one’s physical scope, and self-reflection about the “body”, in terms of the physical and subjective, helps to contextualize life trajectories and their impact [17,18]. Researchers that have used this technique describe that it provides a critical analysis of power social structures, cultural specificities, the intersection of factors related to health and well-being, and relational processes [17,18].

Previous studies have revealed that the body-mapping technique facilitates the expression of culture and the representation of the interactions between people of a specific group based on the reflections about their bodies [19,20]. It is described as helpful towards different goals, including research, clinical, educational, anthropological, therapeutic, and political [19,20]. Other benefits attributed to this technique include being a kind and respectful way to collect data, discussing taboos and complex matters sensitively, clarifying ambiguities, and extensively reflecting on memories, symbols, words, and meanings [17,18,19,20]. Overcoming cultural, linguistic, and educational barriers and promoting awareness about lived experiences are other benefits associated with this qualitative technique [17,18,19,20].

Despite body-mapping having been used in data collection and intervention for vulnerable women in some previous studies [16,18,19,21], the condition of having a mental disorder or experiencing psychosocial distress, as well as living in an economically vulnerable community in Brazil, provides an additional uniqueness to the present study. Furthermore, it is necessary to expand the array of studies that examine this technique and its usefulness in research. We believe that this approach allowed us to focus on important gender and psychosocial issues and explore the meanings of physical and subjective experiences through women’s analysis of the perception of their bodies.

The transferability of the results in the present study is limited given the method used and the fact that it has been developed in a specific region of Brazil. However, this choice made it possible to concentrate on a particular psychosocially vulnerable population culminating in meaningful discussions about the interrelationship between gender issues, mental health, stress, and the body. We understand that these discussions can be helpful in other contexts worldwide, especially in low- and middle-income countries, considering that the sociodemographic characteristics of our sample reveal a social context of vulnerability and poverty found in other cultures.

## 2. Materials and Methods

### 2.1. Study Type

This narrative research consists of a qualitative research approach in which history itself is the data [22,23]. It is intended to explore the experiences lived by the person throughout the story told by them, which makes it possible to construct the meaning of the lived experience [24]. It presupposes that individuals attribute meanings to their experiences and reveal themselves in their own stories [22,23]. Narrative research is composed of three main aspects: temporality (considers the continuity of the experience and the chronological importance of the report), sociality (emphasis on the social context, culture, and environment that influence the experience), and the place where the events took place [22,24]. In this way, the focus is on the chronological sequence of events of people’s experience and their consequences [22,24]. In this approach, storytelling is considered an inherent human capacity, a way of feeling heard, establishing coherence between episodes, and articulating past, present, and future [22,23,25]. Thus, the narrative research process seeks to recognize the multidimensionality of the experience, placing it in a specific “temporal space” [22,25].

### 2.2. Theoretical Framework

The body as a central aspect to experience description permeated all of this study’s development and was conceived based on Donald Woods Winnicott’s propositions about the body and psychosomatic integration [26,27,28,29]. In this theory, the notion of “body–environment” presupposes that the body is a “body of experience” of the relationships it establishes and that reflects a creative contact with a dynamic, human, and alive environment [28]. In this way, the body and the personality compose a cohesive unit. The individual’s relationship with their body reflects a direct connection with their experiences in the environment, demarcating limits, transformations, and circumstances imprinted on it. As physical manifestations are strongly connected with the individual’s history and experiences, the Winnicottian idea of the “body of experiences” emphasizes the importance of studying human nature focusing on the body, considering its interconnections with the environment, relationships, and the meanings attributed to it [26,27]. Therefore, the individual’s perception of their own body reveals its nature, the potential, limits, and particularities of each stage of their life [26,27,28,29]. In this sense, the theory converges with the proposal of the body-mapping technique may enable the participants’ narratives, stimulating the glimpse of their experiences and perceptions considering the main stages of life and emphasizing the correlation between physical manifestations and emotional manifestations as tensions suffered and perceived by them.

### 2.3. Study Setting

This study was carried out from October 2015 to December 2016 in a primary healthcare unit in an inner-city area of São Paulo, Brazil. The estimated population covered by this unit during the study period was 14,148 people. Young adults (25 to 39 years old) make up most of the population in the region, which includes disadvantaged areas for health and social outcomes due to poverty, low levels of education, poor sanitation, and high levels of violence. In this population, 51.4% (7272) were female. The age ranges for female participants were between 0 and 19 years old (29.5%, 2145), 20 to 64 years old (65.3%, 4752), and 65 to 94 years (5.07%, 369).

### 2.4. Participants

Women were invited to participate in this study from a waiting list of 40 women who needed mental health counseling due to a mental health disorder or psychosocial distress. The health unit coordinator provided us with this list. The inclusion criteria were age ≥18 and residing in the neighborhood covered by the above health unit. All women from the list met the inclusion criteria.

The invitations were extended during face-to-face home visits. The study’s objective, the number, location, and duration of the meetings, and the activities that would be developed in each of them were explained to potential participants. A total of 29 women accepted participation into the study. Of the eleven women who refused to participate, eight women claimed they did not have time or were not interested in group activities. Three women did not provide any specific justification.

Participants who had attended less than three focus group sessions (*n* = 15) were excluded from the analysis for this paper because these absences compromised the steps to the development of body maps and consequently the information provided. Thus, the final sample was comprised of fourteen women who met the inclusion criteria, accepted the invitation, and completed the study.

### 2.5. Data Collection

Data collection was performed through focus groups and interviews. Each focal group was developed through four meetings, conducted in a private room of the health unit, and were audio-recorded. These groups included approximately five women in each. The body-mapping technique guide was used as the script for focus groups [16]. This guide was composed of the following topics: representation of marks (physical or subjective) on and under the skin, representing the main stressors in their daily life; drawing their self-portrait as a way to express how they perceived themselves; and memories related to childhood, adolescence, and adult life, as well as social support structures perceived. The dynamics included the stages of the drawings interspersed by discussions and clarifying the meanings attributed by the participants to the pictures, figures, and symbols used in their body maps. The guide was previously tested with three women from another community.

The interviews were made after the four focus group meetings, individually, one with each participant, and guided by the following question: “Considering your body-map, tell me more about your life, emphasizing the aspects that you think that contributed to the more stressful situation that you experience at the moment”. Through this technique, the participants could, in a more detailed way, tell us about their lives as a chronologic process emphasizing the factors that contributed to the recent mental disorder or psychosocial distress. Two female facilitators (one master’s-level and one doctoral-prepared nurse with experience in mental health assistance) conducted the four focus groups, each of which lasted for approximately two hours. The second facilitator performed the interviews. All focus group meetings had a female participant observer (one master’s-level prepared nurse trained in qualitative data collection) who made field notes. The researchers involved in data collection underwent an intensive 90-hour course in qualitative data collection in a graduate program at the University of São Paulo and were unknown to the participants.

### 2.6. Data Analysis

Considering that the adopted theoretical framework also emphasizes the importance of life experiences to the individual’s psychosomatic organization, to their self-perception, and to their ways of expressing themselves in the world [26,27,28,29], the analysis of body maps was based on the description made by the participant about the meaning attributed by her to symbols, figures, and the body represented in the map. This description was made during the focus group meetings and recorded into the field notes. It was understood that this procedure valued the participants’ narratives (an essential aspect for qualitative studies) as well as made it possible, in a way, for them to consolidate their narratives and participate in part of the data analytical process.

Data from focus groups, interviews, and field notes were transcribed, and two nursing researchers conducted content analysis manually, following the steps suggested by Graneheim and Lundman [30]. Successive readings were carried out, at first, to become familiar with the material. Then, more focused reading was undertaken to highlight essential aspects noted in comments and impressions added next to the text.

The next step consisted of identifying the main ideas that emerged from each minimal part of the text. A short name was given to each of these ideas (coding). Then these excerpts and codes were grouped according to the similarity criterion.

Each set of excerpts also received a preliminary title (categorization), and the groupings were successively expanded to the composition of themes. The criterion of mutual exclusivity was considered for the grouping of contents within each theme so that the excerpts and codes within a theme had some similarity. However, each theme was heterogeneous in relation to the others; the themes were mutually exclusive. Considering the importance of the sequential character of the events in the narratives, the “context”, “consequences”, and “future projections” perspectives were prioritized for ordering the themes.

The analytical process was made by both analysts individually (the primary authors). Both researchers compared their interpretations to ensure accuracy and reliability. In all cases, the agreement between both analysts was >80%. Another team member, who had experience with mental health assistance to women, immersed herself in the data and its categorization to inspect and subsequently confirm the findings processed by the analysts. The team then collaboratively discussed and reconceptualized the final categories and themes. The three codes are presented in Table 1.

Because the data collection was carried out in Portuguese, the researchers from the Brazilian team translated the transcripts considering local expressions and idioms. They discussed these with the two foreign researchers on the team, performing the back-translation to ensure an exact match to the original document to reduce any potential bias. The researchers who carried out the fieldwork had proximity to the phenomenon and the local community because they developed teaching activities in the region in previous years and shared the culture and challenges that permeate the daily lives of Brazilian women. This proximity was a positive factor in terms of reflexivity since it facilitated the right to enter the research field. While it may also have been a possible source of bias in interpreting and analyzing data, the collaborative nature of the research group in the analytical phase, appreciation of the interpretation of women themselves about their body maps, and the fact that the group has four researchers outside the field (two of them from other countries) contributed significantly to an analysis broader in terms of the interpretive spectrum. They were configured in the main strategies to deal with possible biases.

### 2.7. Ethical Aspects

The Ethical Committee approved this research of the College of Nursing from the University of São Paulo at Ribeirão Preto (protocol 00876012.2.0000.5393). Participation in the research was voluntary, and, following current Brazilian regulations, the participants did not receive any financial compensation for their participation. They were assured of the confidentiality of the information and that the data would be disclosed only for scientific purposes in journals and academic events with the guarantee of their anonymity. The researchers who performed the data collection have training and experience in the field of mental health and were able to welcome the participants in the face of any emotional reactions during and after the data collection, as well as to facilitate referrals to specialized services with the health team in the case that it was necessary.

## 3. Results

### 3.1. Participant Characteristics

The age of the participants ranged from 21 to 61 years (mean = 44.07, SD = 11.62); the majority were married, had a low level of education and low family income, and declared themselves to be white. They reported mental disorder diagnoses, interpersonal relationship problems, and/or violence in their domestic environment as the main current stressors (Table 2).

Sociocultural factors contributed to the perpetuation of daily difficulties (theme 1). Such difficulties impacted women’s self-esteem and the well-being of their children and contributed to negative feelings and symptoms related to the mental health of these women (theme 2). Participants also referred to their perception of some social supporters and identified several important positive individual attributes that helped them deal with daily difficulties (theme 3).

### 3.2. Family, Cultural, and Socioeconomic Vulnerabilities

Issues related to education, such as low schooling, functional illiteracy, and learning difficulties, were some of the vulnerable conditions reported by the participants. One of them mentioned, “*I had difficulties and constraints learning to read and write. It was difficult to understand the lessons during my school years*” (ID 2). One participant (ID 3) said she did not know how to read, and her nine-year-old daughter helps with the reading when necessary (field note).

Financial difficulties, accompanied by the belief that the man should be the financial family provider, were common among participants. One participant (ID 4) mentioned that she was sad because her husband was not taking responsibility for expenses as she expected. This woman mentioned: “*the man is who should provide the money of the house*”.

All married participants mentioned financial dependency. One of them reported that financial problems were their primary motivation to become married: “*I got married a year after knowing my husband; I got married to leave my parents’ home, to get out that poverty situation*” (ID 14). Another married participant shared her discontent with her financial dependency: “*I do not like to depend on my husband for everything, to have to ask for everything from him. I would like to have my money by myself*” (ID 11).

**Figure 1:**
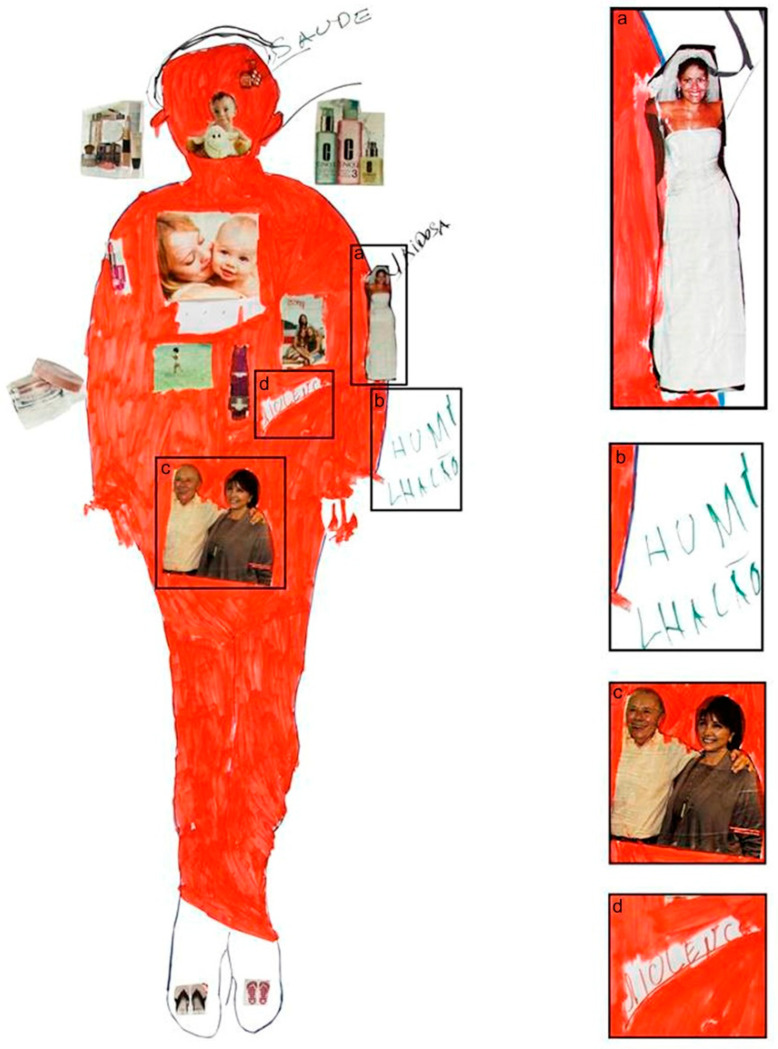
Body map of the participant ID 8. According to this participant, the bride represents her wedding as the most important event of her adult life (**a**). The body was colored red, which she said represented the violence she suffered and was also represented by the word “humiliation” (**b**). Her parents were highlighted as supporters (**c**), and the word “violence” was highlighted inside her body (**d**). Note: Participant used pictures from Brazilian magazines such as “Claudia” and “Veja” to illustrate her body map.

Women described their weddings as one of the most important event of their lives, even those women who referred to domestic violence or gender discrimination in these relationships. For example, one woman (ID 8) said: “*My wedding was the most important event in my adult life*”, and she chose the image of a bride and groom to represent it but took the groom out of the picture and pasted the bride alone on the body map (Figure 1).

Participants mentioned humiliation, threats, and physical and psychological violence: “He (her husband) always called me “sick” and “useless” and said that if it were not for him, I would not have gotten out of that hell that was my parents’ house and that I owed my life to him” (ID 14). Another participant also said that her husband was violent, constantly screaming and humiliating her (ID 8). She wrote the word “violência” (violence) inside of the body map (Figure 1) and the word “humilhação” (humiliation) outside of the body map (Figure 1b). She subsequently asked for red paint and painted the whole body red, leaving only the feet and the word “violence” blank.

### 3.3. Influences of Stressors on the Living Conditions of Women

Conflictual and violent situations permeated the participants’ narrative. They emphasized the consequences of these events on their self-esteem. One of them mentioned that low self-esteem was a horrible thing*;* “*…all the world can tell you that you are beautiful, but you do not believe it*” (ID 14). Another participant (ID 12) cried when she saw her image reflected in the mirror and said she did not like what she saw (field note).

Participants also referred to the influence of the stressors on their sexual life. One woman (ID 8) said that she had not had a sexual relationship with her husband for eleven years. Another (ID 2) mentioned that, since she started treatment for depression a year ago, she had no desire for a sexual relationship with her husband.

**Figure 2:**
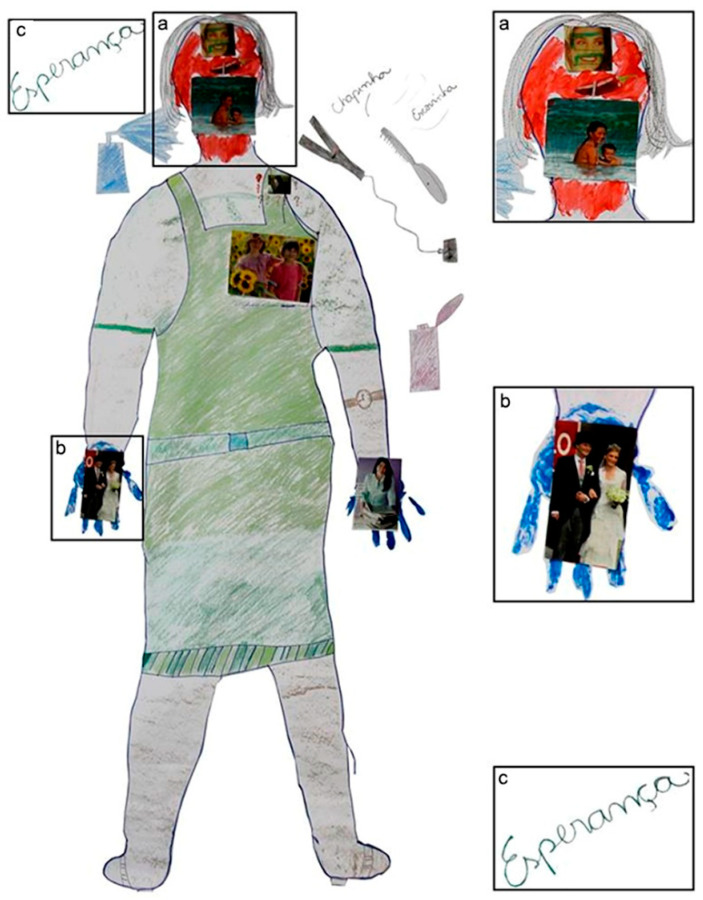
Body map of the participant ID 2. She painted her head in red (**a**), stating it represented “jitters”. In her right hand, she placed a picture of a happy couple to represent her wedding as the most important event of her adult life (**b**); the word “esperança”, “hope” in English, represented her expectations about the future (**c**). Note: The participant used pictures from Brazilian magazines such as “Claudia” and “Veja” to illustrate her body map.

Women also expressed negative feelings such as abandonment, nervousness, and hopelessness. For example, one woman said: “*Even having a family, I always feel abandoned*” (ID 14). One participant painted the head of her body map in red (Figure 2) and explained: “*It is to symbolize my jitters*” (ID 2).

Additionally, most participants mentioned the influence of stressors on their children. Some comments illustrate it: “*My kids are suffering with all this*” (ID 7); “*some days, my younger son has to interfere to stop our fight*” (ID10); “…*my children always witness our discussions, I feel so embarrassed about it*” (ID13). Participant ID 6 reported that her son verbalized that everything was better when his father was no longer at home (field note).

### 3.4. Positive Attributes to Coping with the Stressors

A vital resource highlighted by participants was perceived social support, particularly the support of family members. Some related comments were: “*I have my daughter as a supporter; she and I talk a lot*” (ID 14). “*My mother is very important to me*” (ID 13). “*I call to my son, he comes to me, and I always confide in him*” (ID 11).

Despite all of the reported negative aspects of their experiences, women described some recent events that suggested their determination to seize promising opportunities, especially those related to professional education. The following quote illustrates it: “*I’ve been participating in two free courses for some weeks now, a sewing course and another on hairdressing, to not stay at home*” (ID4). Another participant (ID 9) reported that she was taking a computer course and finding other free courses because it was an opportunity to get out of her house (field note).

**Figure 3:**
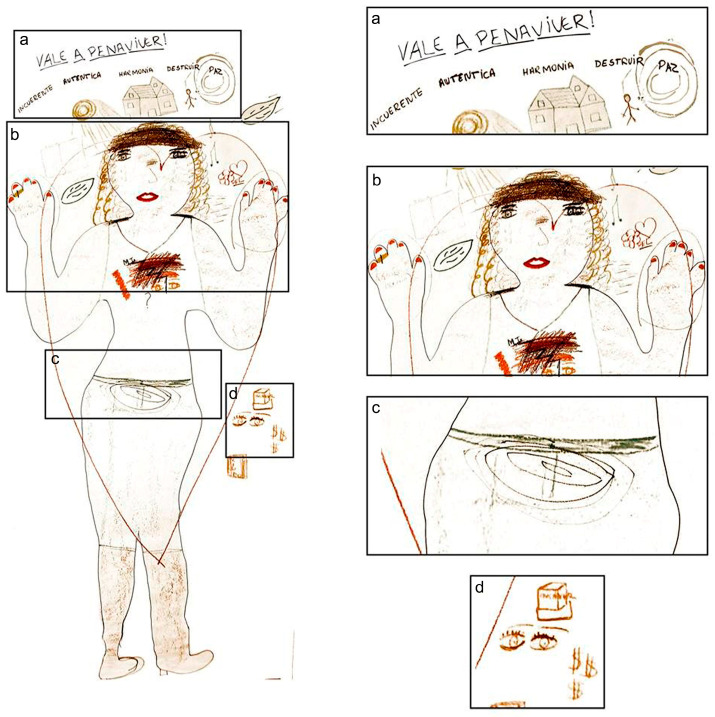
Body map of the participant ID 4. A large heart surrounding her body represented her “need to love herself”. The words on the top of the map express how she perceived herself: “incoerente, autêntica” (incoherent and authentic), and the phrase “vale a pena viver” (“It is worth living”), denoting her hope relating to the future (**a**). She also drew a house and used the word “harmonia” (harmony), representing herself and a man, and the word “destruir” (destroy), representing her need to rebuild herself (**a**). The word “paz” (peace) was in a globe symbolizing the world (**a**). The dark traces in her chest were explained as a broken heart related to her conflictive relationship with her late mother (**b**); in her abdomen, the five uteruses represented her five children (**c**); dollar signs represented her financial problems; the box and book represented a desire to study; the open eyes represented her “need to look to herself” (**d**).

Another positive aspect identified in women’s comments was the ability to establish goals and plans, as observed in the quote: “*Regarding my future, I would like to keep a closer look at myself, at my interior*” (ID 4). This participant represented this concept in her body map with two eyes close to her heart and two outside her body (Figure 3).

Their ability to recover good memories was also a positive result identified during data collection. This ability can be observed in the reporting of one participant about her childhood: “*I used to feel good, I used to feel wanted, a wanted child*” (ID 11). Another also emphasized her family environment during her childhood*:* “*I used to really enjoy staying with a lot of people from my family, laughing, playing, and having conversations*” (ID 5). One participant (ID 1) pasted a photo of a woman holding a child and enjoying the beach. She told us about the first time she saw a beach with her mother, highlighting this episode as a significant memory from her childhood.

Women identified activities that contributed to their well-being and the positive qualities in themselves, as illustrated in the statement: “*I like to do physical activities. I am organized and hardworking*” (ID 9). Another participant told us: “*I really admire my courage*” (ID 14). One woman (ID 1) also recognized that she had achieved many of her dreams and that she was helpful to other people (focus group field notes).

The women expressed their hopes and desires related to work and study. One of them said: “*I would like to study and work*” (ID 4); she represented it in her body map by drawing a book and a box. To complete her body map, she wrote the message: “*Vale a pena viver*” (“It is worth living”) (Figure 3).

## 4. Discussion

### 4.1. The Context of Life, Daily Stressors, and Their Consequences

The main stressors mentioned by the participants were contextualized by a low education level, low family income, and financial dependence, which are also pointed out by previous studies as crucial risk factors for mental disorders and violence [9,10,11,31]. These results reemphasize that the female gender is a critical factor that increases the vulnerability to poverty and precarious mental health conditions [31]. It is essential to highlight the pervasive social and gender inequality, especially in low- and middle-income countries, such as Brazil, which contribute even more to perpetuating these conditions.

The meaning attributed to the experience of marriage in the participants’ life trajectory, even in the case of violence or abuse, raised the discussion about marriage as a socially constructed entity [32]. Typically, it acts as a macro-sociological process, but in this case, it acts on the micro-sociological level as a social process primarily affecting the individual. Considering how marriage is culturally constructed, it can afford a byproduct of the realm of opportunities available to vulnerable women (such as our study participants). In this case, marriage was seen as a “social arrangement that creates for [this] individual the sort of order in which [she] can experience [her] life as making sense” [33].

Thus, many times, women assume the position of invisibility, presenting themselves as figures with no socially valuable attributes. In this way, narratives demarcate feelings of abandonment, hopelessness, low self-esteem, and loneliness, besides the discomfort to depend financially on their partners. In this sense, the longstanding social order in Western countries has legitimized the devaluation of the woman as “appropriate” and “inevitable”, attributing the role of productive and paid work to men and the role of being married, having children, and maternity to women, constituting her as a dependent with her citizenship guarded by masculine figures [5]. Some women still share the logic of dividing roles based on gender, even when they suffer the consequences of this condition, as in the case of some study participants.

The life trajectory narrated by these women, in their perception of their current condition, in general, also referred to the idea of demoralization, understood as difficulty in coping or a discouragement manifested by feelings of impotence, isolation, and despair [34,35,36,37].

Participants mentioned that situations, such as threats and humiliation, were critical aspects of the psychological dimension of violence [10] and corroborate previous research [8,9] that highlighted the direct and indirect impacts of violence in the physical and mental spheres. Given that women do not commonly mention these issues during health service appointments, health professionals should provide a warm and caring environment that allows these women to feel comfortable in sharing these experiences, mainly in the primary healthcare setting, which, in general, are a more accessible healthcare service for vulnerable populations.

Women also reported that violence by their partners affected their children, as also mentioned in previous corroborating studies [9,38]. Some authors have described that approximately 28% of violent events occur in the presence of children and that witnessing this type of event increases the risk of being assaulted, suffering serious psychological disturbances, or even becoming a perpetrator or victim of violence in their adult life [9]. Previous research has also shown that children’s exposure to this type of situation constitutes a type of child abuse, and mothers exposed to violence from their partners have many stressors that can lead to exhaustion, exposing them to the risk of also perpetrating violence on the children [38].

It should be noted that mental distress symptoms were referred to in the narratives of body maps as one of the impacts of the difficulties experienced by these women throughout life, revealing the psychosomatic character of their experiences and corroborating the theoretical framework of the present study.

### 4.2. Resources, Hopes, Desires, and Future Projections

Participants mentioned social supporters, and these were a vital protective factor against violence and its consequences and a mechanism that reinforced self-esteem, self-efficacy, and helped to increase access to health information and social-protection devices [9,11,39].

Summarily, women presented a series of attributes related to good mental health and resilience as a predisposition to accessing available resources, establishing goals and plans, visualizing possibilities, listing positive qualities perceived in themselves, recovering good memories, and identifying activities that provide them with a sense of well-being. In addition, they had important insights related to their situation. These positive aspects assume great relevance when considering mental health encompasses the ability of the individual, group, and environment to interact with others to promote subjective well-being and develop cognitive, affective, and relational skills [31].

Thus, despite the demoralization they felt because of their stressors, the narratives also allude to the concept of “meaning in life” [34,35,36,37] because they reveal adaptive resources and suggest that these women had sought to integrate the conditions imposed on them, to reframe their experiences of suffering, and to give a new direction to their purpose [34,35,36,37].

Paid work and study were the participants’ most immediate desires and possibilities. The extant literature has emphasized that strengthening options and opportunities outside the home context, such as work and study, reduces the risk of experiencing violence perpetrated by their partners, enhances the freedom of mobility, and extends social networks [40]. Furthermore, education can increase the bargaining power of these women in their contexts and increase their knowledge, skills, and resources to make choices that improve their well-being [40]. Despite this, it is recognized that formal education and work are not sufficient to improve self-concept [41] or to change society’s attitudes [40]. There are critical determinants of the mental health of these women that were beyond their control, such as the determinants of health and economic, legal, and environmental factors [31]. Thus, it is necessary to increase their access to information, provide them with practical support services (legal, medical, and police), and raise family awareness about such services, as well as to intensify media campaigns to combat stereotypes [42] and provide spaces to facilitate the discussion and analysis of gender and power [2].

### 4.3. Body-Mapping as a Facilitator of Storytelling

Regarding the role of the body map in the development process of this research, it is worth highlighting both its potential to enable storytelling using verbal, artistic, and visual languages and its relevance concerning the requirements for data collection in the narrative research.

The activity proposal provided here, using figures, drawings, symbols, and colors in the focus groups, sounded to the participants as a somewhat playful technique and less dense than the verbal report alone. This perception was observed mainly during the interactive processes that permeated the development of body maps. Women smiled, joked about their condition, emphasized their artistic skills, and commented on their body shape and performances in their papers’ daily exercise.

For some of them, difficulties with manual skills and insecurity about the aesthetic result of their maps generated some anxiety that was reported at the beginning of the activity. In this sense, the researchers focused not on the aesthetic aspect of the map but the symbolic character of the visual representations and the emphasis was on their meaning to the outlined drawings, figures, symbols, and phrases.

It was identified that the stage of development of the body maps themselves was the “lightest” of the entire process. In contrast, the individual reports during the interview stage were permeated by moments of broken speech, grief, and even crying in some cases. Despite this, these moments were crucial to complement the data collection and for participants to consolidate the narrative of their experiences in detail. These moments also allowed the researchers to give empathy and emotional assistance when necessary.

The stories obtained through the development of body maps, and the report on the meanings attributed to them, formed a dense body of data that allowed in-depth exploration of experiences, considering the logic of the chronological process, and encompassing related aspects such as the relationships, culture, and values of these women. In addition, the outcome proposed by the body map script helped women consolidate the different moments of their life trajectory and envision themselves in the future, as shown in the results.

Thus, we understand that the body-mapping technique is a viable and effective technique to facilitate the storytelling of vulnerable women about the stressors in their daily lives, relating them to their background, reflecting on the consequences in their context and relationships, and the impact upon their own body. Besides being a sensitive and accessible vehicle for generating research data, body-mapping was also a powerful strategy for these women. It facilitated their ability to re-think their life experiences. This technique allowed peers and facilitators to sensitively receive participants’ self-reflection about the sensations and emotions they experienced during their positive and painful moments. The women mentioned that this experience motivated them to change their routine and that it caused them to think about new life plans.

When the broader context of problems is understood, the individual has more opportunities for change and to adopt positions differing from those that maintain the status quo [2]. That is, the body-mapping process may have promoted insights, stressed the remembrance of their trajectories, and helped women to reflect on their motivation to make cognitive changes and behavioral efforts to manage everyday stressors by avoiding them, reducing them, or mitigating their impact on their lives [15].

Considering the theoretical framework adopted [26,27,28,29], we understood that the body-mapping technique may also have enabled the “psychic organization of the subject” and fostered the search for integration between the psychic and somatic parts of the self. We understand that, by enabling storytelling (prioritizing the chronological issue of events, the symbolic re-signification of the experience, and future projections), body-mapping can promote a glimpse of alternatives to the pathological responses that often accompany adverse events. Furthermore, it can encourage the expression of desires, elaboration of plans, and, consequently, expansion of the possibilities of “feeling alive” and “real”.

In terms of practice recommendations, we first point out that all research and practice *must* be situated within broader policies to combat poverty, violence, and gender inequality to be fully effective in dealing with the mental health issues of vulnerable women. Despite this, some insights promoted by this study reemphasize the need to develop specific mental-health-promotion strategies that consider both gender issues and the particularities of cultural life, language, experiences, and health contexts. Moreover, our findings also show that research and practice should promote new meanings of life for participants.

### 4.4. Limitations

This study’s main limitation was the number of participants. Some women may not have felt comfortable sharing their experiences in a group composed of other participants who lived in the same community, which may have been a reason for refusal. We understand that using one-on-one meetings for this technique could attract more participants, however, we valued the impact of the group dynamic. In addition, the minimum number of sessions necessary for the elaboration of the body map reduced the sample size considering the number of absences of some participants.

It is important to emphasize that the low linguistic repertoire of some participants was a challenge to their ability to explain their emotions or describe the meanings they perceived concerning their experiences. Furthermore, thinking about themselves seemed to be an unusual practice among the participants. Thus, we believe that the use colors, symbols, and slogans in the body-mapping technique enabled them to explore and explain themselves more fully. Despite this, not carrying out a specific analysis of the visual material might also be considered a limitation. Finally, reproducing this study in other cultures with a larger and more representative sample is a suggestion for future research.

## 5. Conclusions

The experiences reported by the participants reemphasized a context of socioeconomic vulnerabilities reflected both by the country’s inequalities in terms of access to work, education, and income as well as by gender issues. The stressors most mentioned were related to mental disorders or suffering symptoms, interpersonal conflicts, and violent situations. The impacts of such stressors on the well-being of these women were emphasized, especially in terms of low self-esteem, negative feelings, and demoralization, which was also reflected onto their children. Women revealed resilient traits despite these trajectories marked by suffering and psychosocial precariousness. When reflecting on their bodies from a subjective, environmental, and psychosomatic perspective, they reaffirmed their potential, highlighted their desires, and envisioned new purposes and plans for their lives.

We believe that the body-mapping technique played an essential role in facilitating these women’s storytelling and constitutes a promising tool for enabling self-analysis of experiences and “integrating” them in psychosomatic terms. Moreover, we believe that the technique values the subjective aspects of the experiences; emphasizes symbols and art that facilitate verbalization; promotes reflections related to the present, past, and future; and invites a positive outcome for the subject’s reflection on their own life.

## Figures and Tables

**Table 1:** Themes, categories, and codes from data analysis.

Themes	Categories	Codes
1. Family, cultural, and socioeconomic vulnerabilities	Socioeconomic aspects related to well-being	Issues related to education Financial difficulties Financial dependence
	Sociocultural determinants of gender inequality	Preconceived ideas related to gender role Female submission
	Harmful domestic environment	Family/interpersonal conflicts Violent domestic environment
2. Influences of stressors on the living conditions of women	Psychological aspects	Repercussion on self-esteem Negative feelings
	Behavioral and family aspects	Repercussion on sexual life Repercussion on son’s life
3. Positive attributes and coping with stressors	Aspects related to resilience	Predisposition to seize opportunities Ability to set goals and plans Ability to rescue good memories Identify activities that make them feel better Identify good qualities in themselves

**Table 2:** Characteristics and main complaints of the participants.

ID	Age	Race	Marital Status	Children	Level of Education	Family Income (Minimum Wage) *	Work Situation	Primary Complaint **
1	21	White	Single	0	Undergraduate	>2 to 5	Self-employment	Generalized Anxiety Disorder
2	25	Black	Married	1	High school	>2 to 5	Self-employment	Postpartum depression
3	35	White	Married	4	Functionally illiterate	1 to 2	Housewife	Violence in the domestic environment
4	36	Black	Married	5	High school	>2 to 5	Unemployed	Marital problems
5	42	Brown	Single	0	High school	0 to 1	Not informed	Sequel of Cerebral Vascular Accident
6	46	White	Married	2	High school	No information	Self-employment	Difficulty in relationship with siblings
7	49	Brown	Married	2	Elementary school	No information	Unemployed	Violence in the domestic environment
8	50	White	Married	2	Functionally illiterate	>1 to 2	Unemployed	Violence in the domestic environment
9	51	Brown	Married	3	Functionally illiterate	>2 to 5	Retired	Violence in the domestic environment
10	60	White	Divorced	1	Undergraduate	>2 to 5	Retired	Depression
11	51	White	Married	2	Elementary school	>2 to 5	Housewife	Violence in the domestic environment
12	43	White	Married	3	High school	>2 to 5	Retired	Violence in the domestic environment
13	61	Black	Divorced	5	Elementary school	>1 to 2	Housekeeper	Violence in the domestic environment
14	47	White	Divorced	2	High school	>1 to 2	Unemployed	Violence in the domestic environment

* One Brazilian minimum wage corresponded to approximately USD 136.00. ** The main complaint was described based on information from the participants in the first focus group meeting.

## Data Availability

Not applicable.
